# Primiparity, older maternal age, COVID-19 pandemic, twin birth, and winter birth are associated with lower exclusive breastfeeding in Japanese mothers

**DOI:** 10.1038/s41598-025-20766-4

**Published:** 2025-10-15

**Authors:** Ekachaeryanti Zain, Yuichiro Watanabe, Naoki Fukui, Koyo Hashijiri, Takaharu Motegi, Maki Ogawa, Jun Egawa, Koji Nishijima, Toshiyuki Someya

**Affiliations:** 1 Department of Psychiatry, Niigata University Graduate School of Medical and Dental Sciences, Niigata, 951-8510 Japan; 2 Department of Psychiatry, Uonuma Kikan Hospital, Niigata, 949-7302 Japan; 3 Medical Education Center, Faculty of Medicine, Niigata University, Niigata, 951-8510 Japan; 4 Center for Perinatal, Maternal and Neonatal Medicine, Niigata University Medical and Dental Hospital, Niigata, 951-8520 Japan

**Keywords:** Exclusive breastfeeding, Obstetric characteristics, COVID-19, Birth season, Postpartum period, Perinatal health, Epidemiology, Epidemiology, Nutrition, Preventive medicine

## Abstract

Exclusive breastfeeding is essential for the health and well-being of both mothers and infants, but various factors may influence whether mothers are able to sustain exclusive breastfeeding during the early postpartum period. In this study, we explored the relationship of obstetric characteristics, the timing of delivery during the COVID-19 pandemic, and the season of birth with exclusive breastfeeding among Japanese mothers. Data included self-report survey and medical records of 5719 Japanese mothers at 1 month postpartum. We conducted path analysis on parity, maternal age, type of delivery, type of birth, pandemic-related delivery, birth season, and feeding method. The findings of a good-fit model suggested that being a first-time mother, older maternal age, having twins, giving birth during the pandemic, and delivering in winter were all associated with lower rates of exclusive breastfeeding (all *P* = 0.001). Mothers who were younger, had previous childbirth experience, delivered a singleton, gave birth before the pandemic, and delivered in summer, spring, or autumn were more likely to breastfeed exclusively. These findings support the need for targeted breastfeeding interventions that address obstetric, seasonal, and pandemic-related risk factors.

## Introduction

Research over decades has shown that breastfeeding and human milk are a normative standard for infant nutrition, offering substantial protective factors for mother and infant health^1^. The World Health Organization has developed a global strategy for optimal infant and young child feeding, with exclusive breastfeeding (EBF) for the first 6 months of life, to achieve optimal growth, development, and health^2^. Human milk can facilitate an effective immunological response^3^ and supports metabolism as well as physiological growth^4^ in growing infants. Furthermore, breastfeeding lowers the morbidity and mortality risks related to numerous infectious diseases, such as respiratory infections and gastroenteritis, as well as non-infectious diseases such as allergy, asthma, obesity, and diabetes. Breastfeeding also improves cognitive and brain development in infants^5–7^. In mothers, EBF can promote postpartum recovery and considerably decrease the risk of breast and ovarian cancers^8,9^.

Despite substantial evidence regarding the benefits of breastfeeding, the EBF rate in Japan remains suboptimal, with 54.6% at 3 months^10^ and 37.4% at 6 months^11^. Several factors including obstetric characteristics may affect whether postpartum women practice EBF for their infants^11^. Lower rates of EBF may be associated with primiparity^12–14^, maternal age 35 years or older^15,16^, cesarean delivery^17^, and multiple births^18,19^.

The coronavirus disease 2019 (COVID-19) pandemic resulted in unprecedented challenges that may have further affected breastfeeding practices and support^14,20^. Quigley et al. reported that the frequency of women who practiced EBF at 6 weeks decreased slightly during the pandemic, from 40.9% to 38.2%^20^. However, other obstetric factors were much stronger determinants of these outcomes, including primiparity and cesarean birth^20^.

Seasonal variations have been suggested to affect the EBF rate in several developing countries^21–25^. Two studies in developed countries (the United States and Sweden) reported an association between breastfeeding and the season of birth^26,27^. However, these previous studies only considered the interaction between the birth season and EBF; additionally, research on this association in Japanese mothers is lacking.

Despite the well-documented associations between each of the abovementioned factors and EBF, no studies have thoroughly investigated the relationships between these factors and EBF. In addition to the challenge in implementing a global strategy for infant and child feeding^2^, poor feeding is a serious obstacle to maintaining healthy infant growth and, ultimately, a serious threat to the social and economic development of a country. This highlights the importance of examining multifaceted determinants that contribute to the low rate of EBF among postpartum mothers. Therefore, in this study, we used structural equation modeling (SEM) to examine how multiple factors are associated with the EBF rate among Japanese mothers at 1 month postpartum. SEM allows for a more complex, multidimensional, and precise analysis of empirical data and has been widely applied in the medical and health sciences to test theories involving multiple variables^28^. We proposed a model derived from our hypothesis that parity, maternal age, type of delivery, type of birth, pandemic-related delivery, and birth season can predict the feeding method practiced by postpartum mothers. The findings in this study may lead to recommendations for health care providers, stakeholders, and policymakers to help design effective strategies to improve the EBF rate while considering multifactorial determinants. Understanding such multifactorial determinants will consequently result in recommendations to address challenges related to poor feeding practices among newborns and infants, especially among high-risk groups, to advance optimal maternal and infant health outcomes.

## Methods

### Ethics statement

This study included human subjects and adhered to the latest version of the Declaration of Helsinki. The study was conducted in collaboration with the Department of Psychiatry and Department of Obstetrics and Gynecology at Niigata University Medical and Dental Hospital, along with 33 obstetric institutions across Niigata Prefecture, Japan. Approval for the study was granted by the Niigata University ethics committee (approval number: 2016–0019) and by the ethics committees of the involved obstetric institutions. Before participating in this study, all participants provided their written informed consent after undergoing a detailed explanation of the study. Confidentiality and data protection procedures were strictly followed in accordance with ethical guidelines.

### Participants

The present study was a component of the Perinatal Mental Health Research Project, which was conducted from March 2017 to March 2021^29–38^. This study involved 5719 healthy Japanese mothers who completed a self-report survey during the first month of the postpartum period. Study participants were recruited from 34 affiliated obstetric institutions in Niigata Prefecture, Japan. Participants with severe physical illnesses or obstetric complications requiring hospitalization of the mother or neonate after delivery were excluded. Participants undergoing treatment for psychiatric disorders, as documented in their medical records (e.g., autism spectrum disorder, schizophrenia, major depressive disorder, bipolar disorder, anxiety disorder, and personality disorder), or with physical illnesses that could potentially affect the participant’s mental status (e.g., endocrine disorders, collagen diseases, and neurological disorders) were excluded prior to data collection. However, women with undiagnosed or untreated mental disorders may have been included.

### Measures

We collected data on obstetric characteristics, such as maternal age, parity, type of delivery, type of birth, and date of delivery. Parity included primipara and multipara, the type of delivery included vaginal and cesarean delivery, and the type of birth included singleton and twin birth. Data on feeding methods were collected for the first month postpartum and categorized as EBF, mixed feeding (MF), or formula feeding (FF), based on the World Health Organization definition and a previous study^2,39^. MF and FF were categorized as non-EBF. EBF refers to providing only human milk with no additional fluids, MF refers to human milk combined with formula milk, and FF entails solely providing formula milk.

On the basis of the date of delivery, which referred to the actual calendar date recorded in the medical records, we classified data related to delivery during the COVID-19 pandemic as pre-pandemic delivery (from March 2017 to 5 March 2020) and during-pandemic delivery (from 6 March 2020 to March 2021). This classification is based on a previous study on the effect of the COVID-19 pandemic on breastfeeding support in Japan^14^.

We also determined the season of birth according to the date of delivery. We categorized winter births as those occurring from December to February. Spring births were defined as those occurring from March to May and summer births as those from June to August. September to November births were designated autumn births. This categorization was determined on the basis of the seasonal characteristics in Japan according to the Japan Meteorology Agency^40^ and is consistent with a previous study in a country with four similar seasons^27^.

### Statistical analysis

We initially used the chi-square test for categorical data, which included parity, type of delivery, type of birth, pandemic-related delivery, and birth season, to compare the characteristics of mothers with EBF, MF, and FF. We conducted analysis of variance that met the normality assumption for the continuous variable of maternal age, with the same comparison aim. We excluded participants with missing data from the analyses. The statistical significance of the difference for both tests was set at a *P* value of < 0.008 according to the Bonferroni correction of six statistical tests.

If the chi-square test revealed a significant difference between the birth season and feeding method, we conducted a post hoc test for the chi-square analysis to identify which birth seasons were significantly associated with lower rates of EBF. This approach, called a residual analysis, was conducted so as to obtain the adjusted residual values^41^. We set the Z-critical value with a Bonferroni adjustment of ± 2.86, according to a study by MacDonald and Gardner^42^. Consequently, an adjusted residual value > 2.86 indicates a significantly higher frequency than other values, and a value < − 2.86 indicates a significantly lower frequency than other values.

We then performed SEM analysis to investigate effects among multiple variables simultaneously within our proposed statistical model. First, we performed a path analysis of factors, namely, parity (primipara vs. multipara), maternal age, type of delivery (vaginal vs. cesarean delivery), type of birth (singleton vs. twin), pandemic-related delivery (pre-pandemic vs. during pandemic), birth season (winter vs. spring, summer, and autumn combined), and feeding method (EBF vs. MF and FF combined). The reference and direction for each variable were set to primipara, younger maternal age, vaginal delivery, singleton birth, pre-pandemic, winter birth, and EBF. To handle missing data, we conducted SEM analysis using multiple imputation, resulting in five imputed datasets. Minimal variation was indicated if the standard deviations of each parameter estimate across the five datasets were < 0.01 across all paths. If the minimal variation was indicated, we retained one of the imputed datasets for reporting. We reported the estimate (*β*) value for each path, which represents the standardized path coefficient, indicating the strength and direction of the relationship between two variables. We used the bootstrapping method with 2000 resamples and obtained 95% bias-corrected confidence intervals (CIs). We then set statistically significant paths (*P* < 0.05) after the analysis. We tested the goodness of fit of the model with two indices, a comparative fit index ≥ 0.95 and a root mean square error of approximation ≤ 0.08^43^.

We conducted all statistical analyses using the IBM SPSS version 31 (IBM Corp., Armonk, NY, USA) and Amos 25.0.0 (IBM Japan, Tokyo, Japan).

## Results

We included all data from the 5719 mothers at 1 month postpartum with complete recorded data on the feeding method (EBF, MF, or FF; *n* = 2704, 2843, and 172, respectively) (Table 1). We excluded mothers with missing values for maternal age, parity, type of delivery, and type of birth (*n* = 18, 35, 44, and 95, respectively) from the analysis.

**Table 1:** Associations between obstetric characteristics, pandemic-related delivery, and the birth season and feeding methods.

Variable	Value	Feeding methods			*P* value
		EBF	MF	FF	
Maternal age (n/years)	5701/32.1 ± 4.9	31.86 ± 4.88	32.46 ± 4.93	31.60 ± 5.44	< 0.001
Parity (n)	5684				< 0.001
Primipara	2692	1028 (32.8%)	1569 (58.3%)	95 (3.5%)	
Multipara	2992	1659 (55.4%)	1256 (42.0%)	77 (2.6%)	
Type of delivery (n)	5675				0.054
Vaginal delivery	4712	2262 (48.0%)	2317 (49.2%)	133 (2.8%)	
Cesarean delivery	963	425 (44.1%)	503 (52.2%)	35 (3.6%)	
Type of birth (n)	5624				< 0.001
Singleton	5564	2650 (47.6%)	2748 (49.4%)	166 (3.0%)	
Twin	60	12 (20.0%)	46 (76.7%)	2 (3.3%)	
Pandemic-related delivery (n)	5719				< 0.001
Pre-pandemic	5001	2414 (48.3%)	2435 (48.7%)	152 (3.0%)	
During pandemic	718	290 (40.4%)	408 (56.8%)	20 (2.8%)	
Birth season (n)	5719				< 0.001
Winter	1351	569 (42%)	732 (54%)	50 (4%)	
Spring	1468	676 (46%)	750 (51%)	42 (3%)	
Summer	1556	801 (51%)	719 (46%)	36 (2%)	
Autumn	1344	658 (49%)	642 (48%)	44 (3%)	

Data (with no missing values) are expressed as the number (percentage) or mean ± standard deviation.

The percentage for each category of feeding method was rounded.

The level of significance was set at an overall *P* < 0.008 based on Bonferroni correction for six tests. Analysis of variance was used for maternal age, and the chi-square test was used for parity, type of delivery, type of birth, pandemic-related delivery, and birth season.

Pre-pandemic was defined as delivery from March 2017 to 5 March 2020, and during pandemic was delivery from 6 March 2020 to March 2021. Winter was from December to February, spring from March to May, summer from June to August, and autumn from September to November. Abbreviations: EBF, exclusive breastfeeding; MF, mixed feeding; FF, formula feeding.

We found significant differences in the mean maternal age (F = 11.30, *P <* 0.001), parity (χ^2^ = 169.4, *P* < 0.001), type of birth (χ^2^ = 18.49, *P* < 0.001), pandemic-related delivery (χ^2^ = 16.74, *P* < 0.001), and birth season (χ^2^ = 30.62, *P* < 0.001) among mothers with EBF, MF and FF (Table 1).

We subsequently performed post hoc analyses of residual tests with Bonferroni adjustment. With winter birth, the frequency of EBF was significantly lower and that of MF was significantly higher (adjusted residual = − 4.4 and 3.8, respectively) than those with other birth seasons (Table 2). With summer birth, the frequency of EBF was significantly higher and that of MF was significantly lower (adjusted residual = 3.9 and − 3.2, respectively) than those with other birth seasons.

**Table 2:** Post hoc analysis with adjusted residual values for associations between birth seasons and feeding methods (*n* = 5719).

Season of birth	Sample Size (*n*)	Feeding methods		
		EBF	MF	FF
Winter	1351	−4.4*	3.8*	1.7
Spring	1468	−1.1	1.2	−0.4
Summer	1556	3.9*	−3.2*	−1.9
Autumn	1344	1.4	0.7	−1.6

* Statistically significant. If the residual value is > 2.86, it indicates a significantly higher frequency than other values, and if it is < − 2.86, it indicates a significantly lower frequency than other values.

Winter was from December to February, spring from March to May, summer from June to August, and autumn from September to November.

Abbreviations: EBF, exclusive breastfeeding; MF, mixed feeding; FF, formula feeding.

**Fig. 1:**
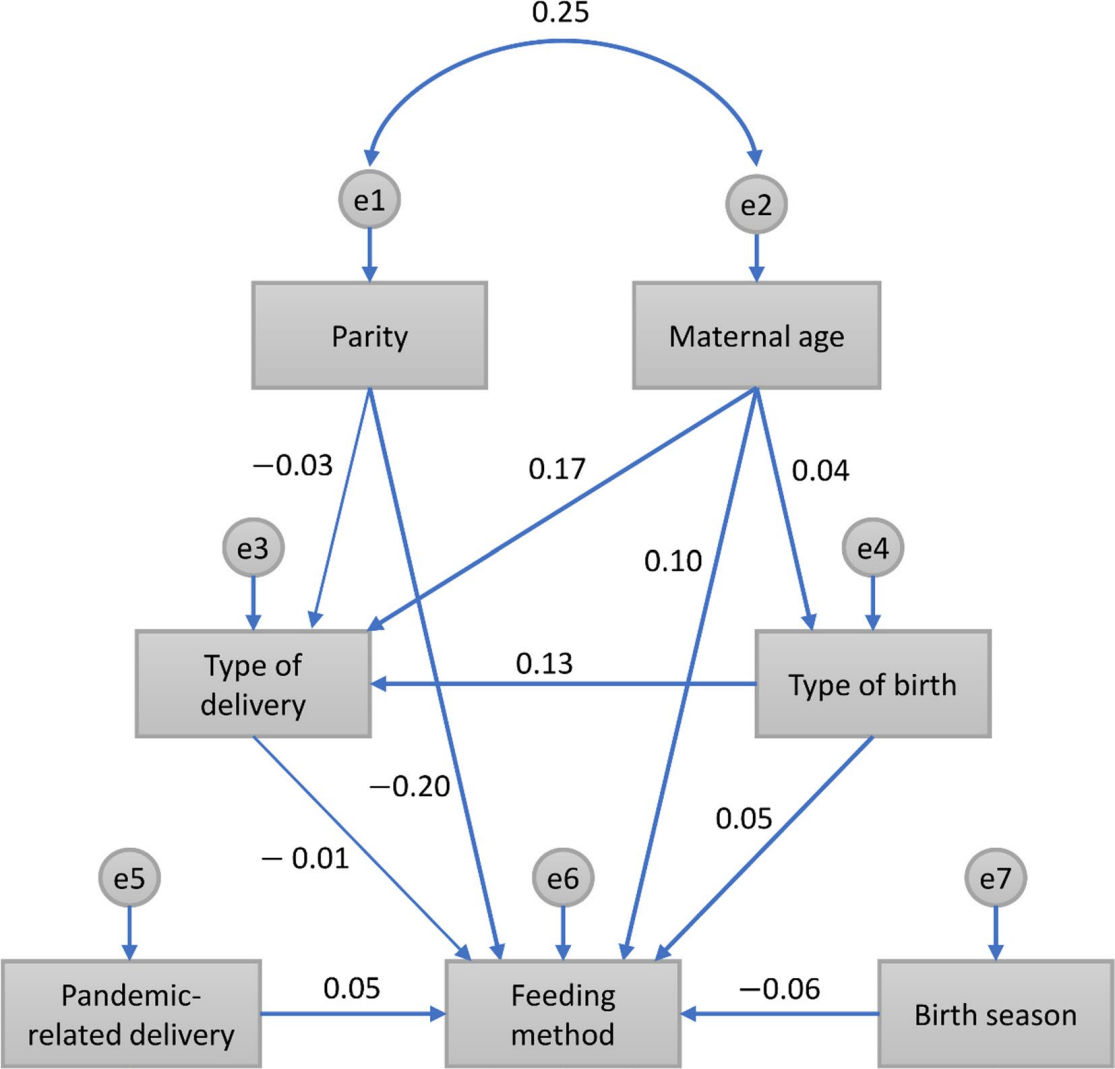
Path model of the association between obstetric characteristics, pandemic-related delivery, birth season, and feeding method. Bold lines showed significant paths (All *P* = 0.001). Parity: primipara (as reference) vs. multipara; younger maternal age (as direction); type of delivery: vaginal (as reference) vs. cesarean delivery; type of birth, singleton (as reference) vs. twin; pandemic-related delivery: pre-pandemic (as reference) vs. during-pandemic; birth season: winter (as reference) vs. spring, summer, and autumn combined; feeding method: exclusive breastfeeding (as reference) vs. mixed and formula feedings combined.

We performed SEM analysis with bootstrap for each imputed dataset, indicating minimal variation in parameter estimates with standard deviations < 0.002 across all paths. Therefore, the results from the first imputed dataset were retained for reporting. From this dataset, a path analysis revealed the associations between feeding method and obstetric characteristics, pandemic-related delivery, and birth season (Fig. 1). The path model was an excellent fit for the data (comparative fit index = 0.977 and root mean square error of approximation = 0.019). In the path model, the five factors of parity, maternal age, type of birth, pandemic-related delivery, and birth season showed significant direct effects on predicting the feeding method (all *P* = 0.001, based on 2000 bootstrapped samples). Specifically, according to the relationship direction and reference of each variable, primiparity (*β* = −0.20; 95% CI − 0.222, − 0171), older maternal age (*β* = 0.10; 95% CI 0.075, 0.129), twin birth (*β* = 0.05; 95% CI 0.026, 0.071), during-pandemic delivery (*β* = 0.05; 95% CI 0.030, 0.077), and winter birth (*β* = −0.06; 95% CI − 0.08, − 0.03) had significant direct effects on predicting lower rates of EBF practices (Table 3). Among these, parity and maternal age had the largest standardized path coefficients (*β* values), indicating a relatively stronger association with feeding method compared with the other factors.

**Table 3:** Direct effect coefficients of multiple variables on feeding methods in the path model.

Model fit	Pathway	Estimate (β)	95% confidence interval (lower limit, upper limit)	*P*
CFI: 0.977	Parity → Feeding method	−0.20	−0.222,−0.171	0.001*
RMSEA: 0.019	Maternal age → Feeding method	0.10	0.075, 0.129	0.001*
	Type of delivery → Feeding method	0.01	−0.013, 0.037	0.425
	Type of birth → Feeding method	0.05	0.026, 0.071	0.001*
	Pandemic-related delivery → Feeding method	0.05	0.030, 0.077	0.001*
	Birth season → Feeding method	−0.06	−0.08, − 0.03	0.001*

*P-value was significant at *P* < 0.05.

Estimate (*β*) represents the standardized path coefficient.

Parity: primipara (as reference) vs. multipara; younger maternal age (as direction); type of delivery: vaginal (as reference) vs. cesarean delivery; type of birth: singleton (as reference) vs. twin; pandemic-related delivery: pre-pandemic (as reference) vs. during-pandemic; birth season: winter (as reference) vs. spring, summer, and autumn combined; feeding method: exclusive breastfeeding (as reference) vs. mixed and formula feeding combined.

Abbreviations: CFI, comparative fit index; RMSEA, root mean square error of approximation.

## Discussion

The findings of this study showed that primiparity, older maternal age, twin birth, delivery during the COVID-19 pandemic, and winter birth were associated with a lower prevalence of EBF during the first month postpartum. Taking into consideration the load of each standardized path coefficient in the model, primiparity and older maternal age may contribute more strongly to non-EBF practices. Similarly, twin birth, delivery during the pandemic, and winter birth are also associated with higher rates of non-EBF practices, although to a lesser extent. By contrast, the type of delivery did not affect the feeding method in this study population.

The current findings show that primiparity has the most considerable effect on predicting non-EBF. Consistent with our findings, several studies have reported that primiparas had a significantly lower EBF rate than multiparas^12–14^. Primiparity was one factor identified as a predictor of early breastfeeding cessation at 1 month postpartum in a Taiwanese study^12^, which may have been caused by a lack of knowledge and experience in breastfeeding techniques for first-time mothers. Furthermore, a study in Japan reported that the proportion of primiparas who breastfed exclusively at 1 week and 1 month postpartum was lower in comparison with the proportion of multiparas^44^. A study in Denmark further stated that primiparas tend to have doubts about breastfeeding and to feel anxious and depressed within the 6 weeks after birth^45^.

Consistent with our results, a previous study reported that Japanese mothers aged 35 years or older had the greatest risk of failure to initiate EBF, especially when they were also primiparous^15,44^. Moreover, a previous study among Japanese mothers showed that older maternal age was associated with non-EBF^14^. In line with this finding, our study population (with a maternal age range of 27 to 37 years) also showed that younger mothers had a higher rate of EBF than older ones.

In the current study, we found that twin birth significantly predicted a decrease in EBF. A previous meta-analysis reported a lower rate of EBF among mothers with twin or multiple births than among those with a singleton birth because of various factors such as unwillingness to breastfeed or a physical and psychological burden on the mother owing to work–breastfeeding conflicts, complex breastfeeding management, or a lack of support from family and medical professionals^18^. Additionally, although mothers with multiple infants may have a certain determination regarding breastfeeding and consider breastfeeding to be an integral part of their identity as a mother, mental health is often affected by their experiences in facing challenges with care taking and insufficient milk supply, which may be the cause of low EBF in this group^19^.

A main finding in the current study was that, in addition to some obstetric characteristics that contribute to breastfeeding practices, the unprecedented COVID-19 pandemic also had a major role in affecting breastfeeding practices. We found that delivering during the COVID-19 pandemic may have contributed to a decreased rate of EBF. This finding is consistent with a previous study in the United Kingdom that reported an 8% decrease in the EBF rate at 6 weeks postpartum during the COVID-19 pandemic^20^. Another study conducted in Japan also reported a decrease in breastfeeding support during the pandemic^14^, and a study reported that this insufficient support caused a decrease in the EBF rate during the pandemic^46^. Furthermore, a study reported that city lockdown considerably affected the decrease in EBF^47^. Additionally, reduced professional support to promote breastfeeding was prevalent during the COVID-19 pandemic, suggesting that family-centered breastfeeding strategies are necessary^48^.

The main concern affecting breastfeeding during the pandemic was regarding maternal–infant transmission of COVID-19 infection and its effects on neonatal and infant outcomes, especially among mothers with or suspected of having COVID-19^49^. Despite this, an extensive study involving Thailand, the United Kingdom, South Korea, Taiwan, and Brazil on breastfeeding during the COVID-19 pandemic showed that even if mothers had ever tested positive for COVID-19 during the pandemic, they were more likely to breastfeed when they were multiparous and had positive beliefs about breastfeeding^50^. Therefore, the pandemic may have added additional risk for mothers with characteristics that already make them less likely to breastfeed exclusively.

We also found that EBF practice was affected by seasonal variation according to the month of birth. The EBF rate in Japan was lower with winter birth than with other birth seasons. In contrast, a study in the United States showed that the rate and duration of EBF were lower for male children born in the spring than for those born in other seasons^27^. One potential reason for this discrepancy between studies is that mothers in the United States may be more likely to stay indoors during cold weather and may have greater awareness about preventing respiratory infection, which can facilitate more frequent breastfeeding^27^. In the Japanese context, although there is limited evidence on the effect of seasonal outdoor activity on breastfeeding, one study reported that Japanese women who were breastfeeding at 1–2 months postpartum had a higher dietary intake of salad vegetables during summer compared with other seasons^51^. A study among Filipino mothers reported that higher maternal intake of fruits and vegetables was correlated with longer breastfeeding duration^52^. Therefore, the seasonal dietary pattern may offer a potential explanation for differences in the quality and quantity of human milk produced during summer compared with other seasons. This could facilitate more frequent breastfeeding among Japanese mothers in warmer seasons, although further research to clarify this is needed. Another study reported that women who gave birth in the other seasons were less likely to have postpartum depression compared with those who gave birth in winter^53^ may also be the case among Japanese mothers, but further investigation to confirm this is necessary. Although the pattern of breastfeeding rates was found to differ among certain seasons in some countries with four seasons, this evidence indicates that regional seasonality may have an effect on EBF depending on the season when the neonate is born.

Seasonal variation in breastfeeding tends to be overlooked in comparison with other factors. Our study suggested that the lower EBF rate with winter birth in Japan may contribute to a higher incidence of infections during winter, as reported previously^54–56^. This may be owing to a reduction in the immunologic protection that is typically conferred by human milk^57^. Several studies have identified specific nutritional components in human milk that provide immunologic protection against various infections and may also provide long-term benefits for physical health^57–59^. For example, a study in Sweden reported that children born during summer were more susceptible to developing diabetes mellitus in adulthood^26^. That study further reported that these summer-born individuals were exclusively breastfed for a shorter duration during infancy compared with those born in other seasons. Taken together, these past studies suggest that reduced breastfeeding, particularly during certain seasons, may contribute to seasonal variations in infection or disease risk. Our findings highlight the importance of also recognizing the seasonal variation factor in breastfeeding practices.

Our results suggested that there may be a complex interaction among parity, maternal age, type of birth, pandemic-related delivery, and birth season in association with EBF for Japanese mothers at 1 month postpartum. Health care providers should consider these factors when tailoring breastfeeding support programs. Specifically, when mothers giving birth have risk factors for non-EBF, such as first-time mother, age 35 years or older, twin or multiple birth, or combined risks, additional support and resources might be necessary to encourage EBF of a newborn in the first month of life. Additionally, a more focused breastfeeding support program should be provided to mothers who deliver during the winter season in Japan. Any unprecedented event related to a disease outbreak, such as a pandemic situation, requires special intervention to support breastfeeding. Family-centered breastfeeding education programs may be especially important when health care systems are overwhelmed owing to an outbreak response^48^. Although the SEM model used in this study does not generate predictions at the individual level, it provides valuable insights into population-level patterns. We conducted our analysis to identify trends in a population exposed to multiple factors that may be associated with EBF. Future research should explore the cumulative or synergistic effects of co-occurring risk factors in individual mothers as their combined presence may compound the risk of early breastfeeding cessation beyond the sum of their individual effects. Such investigations would provide a deeper understanding of high-risk profiles and help design more targeted interventions to support EBF.

Stakeholders and policymakers should also consider factors such as obstetric characteristics to support policies that create and sustain a maternal environment conducive to EBF practice. Such policies should include the distribution of accurate information, such as the fact that breastfeeding is superior to FF, as well as support for breastfeeding mothers within the health care system, mothers’ families, workplaces, communities, and support for training programs targeting health care providers, counselors, and consultants in educating mothers regarding knowledge and skills related to lactation. Such support is particularly important for first-time mothers, those aged 35 years or older, and those who have twin or multiple births. Stakeholders should also consider the seasonal variations in breastfeeding and disease outbreaks when designing and implementing strategies to sustain a high breastfeeding rate, where additional solutions to manage obstacles related to the season or a disease outbreak may be required.

### Study strengths and limitations

The main strengths of our study include the large sample size, focus on a specific population, cross-sectional design in which the collection of breastfeeding data was conducted prospectively, and SEM analysis. Nonetheless, several important limitations must be acknowledged. First, breastfeeding was assessed only at 1 month postpartum, which limits the ability to understand longer-term breastfeeding patterns and associated factors. Additionally, we did not collect data on socioeconomic status (e.g., maternal education, employment status, household income), breastfeeding intention, breastfeeding self-efficacy, skin-to-skin contact after birth, perceived support, perceived stress, mental health status, or time availability to breastfeed during holidays or maternity leave. Consequently, although all variables (with the exception of type of delivery) included in our model were significantly associated with EBF, the present findings should be interpreted with caution because unmeasured confounding factors may have influenced the results. For example, maternal education has been identified as a confounding variable for maternal age, and the association between maternal age and EBF may disappear or become substantially weaker when education is controlled^60^. However, the use of SEM in this study allows for the estimation of error variances, which may partially account for the impact of these unmeasured variables. Future research is needed to address these limitations, including studies with longer follow-up periods and investigations involving more diverse populations, to enhance the generalizability of the findings beyond the Japanese context.

## Conclusions

In this study, we found that multiple contributing factors were associated with a lower rate of EBF practice among Japanese mothers. Primiparity, older maternal age, twin birth, delivery during the COVID-19 pandemic, and winter birth were associated with a lower prevalence of EBF in Japanese mothers at 1 month postpartum. Understanding these associations can help health care providers and policymakers tailor strategic interventions to more effectively promote breastfeeding under specific circumstances, ultimately improving maternal and neonatal health outcomes. Future research involving more diverse populations and using longitudinal or interventional designs are essential to explore potential causal interactions among factors contributing to lower and higher EBF rates, thereby enhancing the generalizability and applicability of the present findings.

## Data Availability

The data that support the findings of this study are available from 34 affiliated obstetric institutions in Niigata Prefecture, Japan, as listed in the acknowledgment section, but restrictions apply to the availability of these data, which were used under license for the current study, and so are not publicly available. Data are however available from the corresponding authors upon reasonable request and with permission of the 34 affiliated obstetric institutions in Niigata Prefecture, Japan.
